# T1a期非小细胞肺癌相关预后研究

**DOI:** 10.3779/j.issn.1009-3419.2010.03.04

**Published:** 2010-03-20

**Authors:** 忠吾 胡, 屠阳 申, 征平 丁, 强 谭, 允中 周

**Affiliations:** 200030 上海，上海交通大学医学院，上海市胸科医院，上海市肺部肿瘤临床医学中心 Shanghai Jiao Tong University Medical College, Shanghai Chest Hospital, Shanghai Lung Tumor Clinical Medical Center, Shanghai 200030, China

**Keywords:** 肺肿瘤, T1a, 细胞分化, 分期, 预后, Lung neoplasms, T1a, Cell differentiation, Stages, Prognosis

## Abstract

**背景与目的:**

新的第7版非小细胞肺癌（non-small cell Lung cancer, NSCLC）分期已经提出肿瘤直径对NSCLC预后的重要性，但是对癌灶直径≤2 cm预后研究尚少。本研究旨在探讨影响癌灶≤2 cm（T1a）的Ⅰ期NSCLC病人的临床预后因素，为临床预后治疗提供依据。

**方法:**

回顾性分析本院既往手术后病理诊断为癌灶≤2 cm（T1a）的Ⅰ期的73例NSCLC病人，运用统计学软件SPSS 17.0分析影响长期生存的临床因素。

**结果:**

该类患者5年以上总体生存率达到80.8%。性别（*P*=0.175）、年龄（*P*=0.241）、病理类型（*P*=0.265）、是否行系统纵隔淋巴结清扫手术（*P*=0.918）、是否术后辅助化疗（*P*=0.616）、是否侵犯胸膜（*P*=0.827）和该类患者的长期生存无明显相关性。影响预后的主要因素是癌灶的分化程度，即在中度分化程度以下长期生存较差（*P*=0.01）。

**结论:**

在癌症直径≤2 cm的Ⅰ期肺癌中，侵犯胸膜对生存无影响，而分化程度是一个重要的独立预后因素。

## Abstract (en)

**Background and objective:**

The new edition of the TNM staging for lung and pleural tumours has been finished, which put weight on the extent of primary tumor as one of the important prognosises. But little study has performed on the primary tumor extent ≤2 cm. The aim of this study is to explore the prognosis of patients with tumor extent ≤2 cm in stage Ⅰ of non-small cell lung cancer, which helps us to choose the best treatment for these patients.

**Methods:**

Retrospective study on the clinical response and survival time of whom underwent complete surgical resection and diagnosed as T1a of stage Ⅰ NSCLC from 1998 to 2004 was analyzed. Data was analyzed by SPSS 17.0 software.

**Results:**

Overall survival rate was 80.8%. By the study, age (*P*=0.241), gender (*P*=0.175), history of smoking (*P*=0.845), pathologic type (*P*=0.265), and systematic mediastinal lymphadenectomy (SML)(*P*=0.918) or not, postoperative adjuvant chemotherapy or not (*P*=0.616) and visceral pleural invasion (*P*=0.827) were not the prognosises of these patients. Only the tumor differentiation such as poorly differentiated was the important prognosis (*P*=0.01).

**Conclusion:**

In the tumor extent ≤2 cm of stage Ⅰ non-small cell lung cancer, the visceral pleural invasion maybe not influence the patients survival. The tumor differentiation is one of the important prognostic factors.

肺癌是常见的恶性肿瘤，位居男性肿瘤发病的第一位，女性肿瘤的第二位，每年因肺癌致死约100多万人，并且趋势还在上升^[1]^。早期肺癌的诊断比例较低，大部分病人诊断时已经为中晚期，治疗效果较差。根治性手术、术后辅助化放疗、新辅助化疗等多学科个体化的肺癌治疗模式，使肺癌的的生存有所改善^[2, 3]^。由于国内外对直径≤2 cm早期NSCLC预后研究较少，本研究回顾性分析研究了73例既往诊断为Ⅰ期的癌灶≤2 cm（T1a）的非小细胞肺癌（non-small cell lung cancer, NSCLC）病人的临床资料，结合随访结果，探讨影响预后的各种临床因素，并讨论外科纵隔淋巴结清扫方式，和术后辅助化疗对患者生存意义。现总结如下:

## 1 材料和方法

### 1.1 临床资料

回顾性研究1998年-2004年上海市胸科医院外科收治的手术后病理诊断为T1a Ⅰ期的上海籍NSCLC患者，排除死于其他疾病的随访患者，共收集73例病人。其中男性35例，女性38例，年龄范围34岁-78岁，平均年龄60岁。低龄组（不大于60岁）患者35例，高龄组（大于60岁）患者38例。吸烟者29例，不吸烟者44例。无病体检主诉入院行治疗者71例，其他疾患者查体发现入院者2例。腺癌52例，鳞癌13例，大细胞等肺癌7例。所有患者均行手术治疗，其中行系统纵隔淋巴结清扫者22例，行纵隔淋巴结采样者51例。未侵犯胸膜者28例，侵犯胸膜者45例。Ⅰa期28例，Ⅰb期45例。分化程度在中分化以下者18例，以上包括中分化者55例。术后行化疗者7例，未行化疗者66例（详见表 1）。

**1:** 癌灶≤2 cm（T1a）的Ⅰ期NSCLC患者临床病理特征和生存相关分析 Effects of clinical characteristics on the survival time of patients with tumor extent ≤2 cm (T1a) in stage Ⅰ NSCLC

Characteristics	*n*	Long survival	*P*
Smoking			0.845
Yes	29	23	
No	44	36	
Age			0.241
≤60	35	30	
> 60	38	29	
Sex			0.175
Female	38	33	
Male	35	26	
Mediastinal lymphadenectomy			0.918
SML	22	18	
MLS	51	41	
Visceral pleural invasion			0.827
Yes (Ⅰb)	45	37	
No (Ⅰa)	28	22	
Tumor differentiation			0.010
Moderately and well	55	48	
Poorly	18	11	
Adjuvant chemotherapy			0.616
Yes	7	5	
No	66	54	
Histology			0.265
Adenocarcinoma	53	44	
Squamous cell carcinoma	13	11	
Others	7	4	
SML: systematic mediastinal lymphadenectomy; MLS: mediastinal lymph node sampling; NSCLC: non-small cell lung cancer.			

### 1.2 病例纳入标准所有病人

所有病人入院皆行血液生化检查、肝肾功能检查、颅脑CT检查、骨扫描、上腹B超，排除脑、骨、上腹脏器的远道转移，肺功能测试肺功能状态可耐受手术，无手术禁忌。纤维支气管镜检查了解气道情况，无气道内播散。

### 1.3 治疗方法

所有患者皆行完整手术切除，支气管切断阴性。手术医生根据胸CT影像资料和术中淋巴结肿大情况评估作系统淋巴结清扫或采样手术。系统纵隔淋巴结切除术按Mountain 1997^[4]^年颁布的标准进行。术后根据患者经济能力、主观愿望和内科医生评估，部分病人术后行1到4个疗程的含铂方案的术后辅助化疗。

### 1.4 疗效评价

随访病人的总体生存率（overall survival, OS），截止日期为2009年6月8日。根据患者的生存期，评估≤2 cm（T1a）的Ⅰ期NSCLC患者的临床病理特征、治疗方案对患者长期生存预后的影响。

### 1.5 统计学分析

采用SPSS 17.0版统计软件，进行统计分析，生存率计算采用*Kaplan-Meier*法，运用*Log-rank*检测统计学意义，采用*Cox*回归分析多变量资料与长期生存的关系，*P* < 0.05为有统计学意义。

## 2 结果

### 2.1 生存情况

用*Kaplan-Meier*生存曲线分析73例T1a的Ⅰ期NSCLC，5年内死亡12例，总体5年长期生存达83.56%。整个随访期间死亡14例，长期生存率80.8%（图 1）。

**1:**
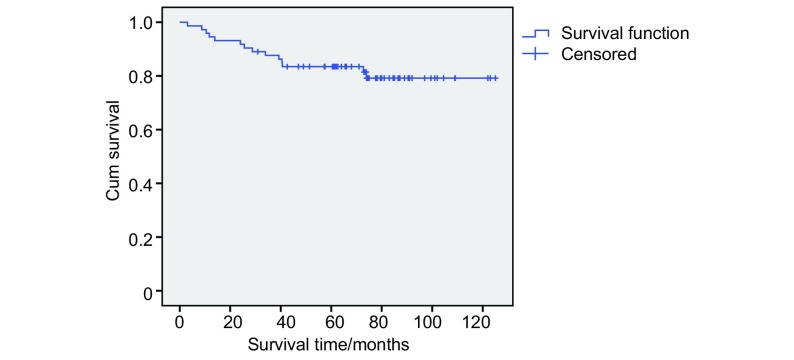
73例癌灶≤2 cm（T1a）的Ⅰ期NSCLC患者的长期生存曲线 Survival curve of patients with tumor extent ≤2 cm (T1a) in stage Ⅰ NSCLC

### 2.2 *Cox*多因素回归模型分析长期生存相关因素

研究发现在T1a的Ⅰ期NSCLC患者的临床病理特征中性别、是否吸烟、年龄高低、是否纵隔淋巴结系统切除、是否侵犯胸膜、术后辅助化疗因素、病理类型、TNM分期和长期生存无相关性，对于T1a的Ⅰ期NSCLC生存并无影响。而仅分化程度影响患者长期生存（95%CI: 1.274-10.371, *P*=0.01）（表 1）。

### 2.3 *Kaplan-Meier*法单因素生存分析

对T1a的Ⅰ期NSCLC病人分化程度影响患者生存，我们将中低分化和低分化归为中分化程度以下，而高分化和中高分化归为中分化程度以上，相关分析可见中分化程度以下患者的长期生存比中分化（包括）以上程度者生存率要低，对长期生存有统计学意义（*P*=0.01）（图 2）。而是否侵犯胸膜（*P*=0.827）（图 3）和系统淋巴结清扫（*P*=0.918）（图 4）并不是长期生存的预后因素。

**2:**
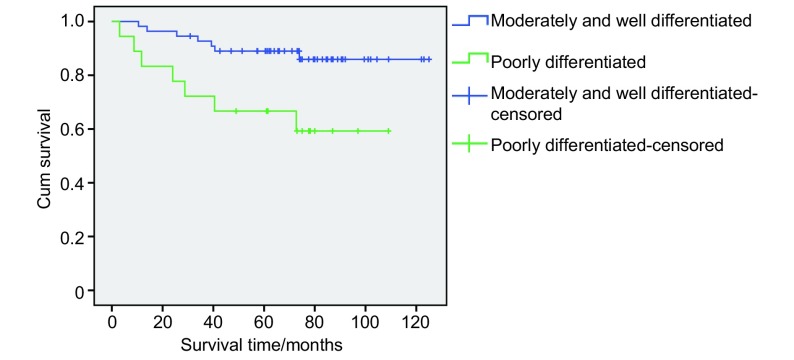
癌灶≤2 cm（T1a）的Ⅰ期NSCLC患者分化程度的生存曲线 Survival curves of the different tumor differentiation of patients with tumor extent ≤2 cm (T1a) in stage Ⅰ NSCLC

**3:**
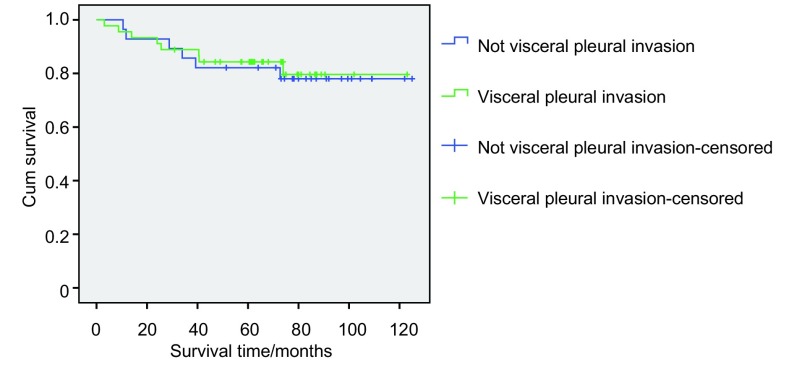
癌灶≤2 cm（T1a）的Ⅰ期NSCLC患者是否侵犯胸膜即分期的生存曲线 Survival curves of visceral pleural invasion or not of patients with tumor extent ≤2 cm (T1a) in stage Ⅰ NSCLC

**4:**
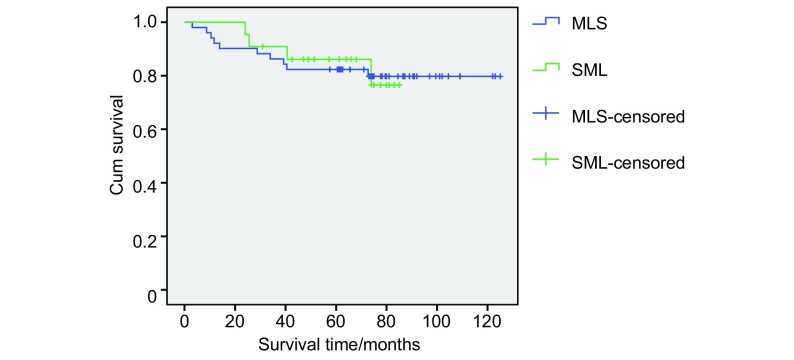
癌灶≤2 cm（T1a）的Ⅰ期NSCLC患者是否系统淋巴结清扫的生存曲线 Survival curves of systematic mediastinal lymphadenectomy or not of patients with tumor extent ≤2 cm (T1a) in stage Ⅰ NSCLC

### 2.4 分化程度是重要的预后

按肺癌细胞分化程度进行分组，中分化以下者，长期生存率有61.1%，中高分化组患者长期生存率87.3%。在细胞低分化组中，2例病人行含铂方案术后辅助化疗长期生存2例，余未行术后辅助化疗的16例患者死亡7例，长期生存是56.3%。中高分化的患者中，5例术后行含铂方案的术后辅助化疗患者，长期生存3例，50例未化疗者，长期生存45例，长期生存率达90%（表 2）。

**2:** 癌灶≤2 cm（T1a）的Ⅰ期NSCLC患者术后辅助化疗对生存的影响 Effects of postoperative adjuvant chemotherapy on the survival time of patients with tumor extent ≤2 cm (T1a) in stage Ⅰ NSCLC

Tumor differentiation	Adjuvant chemotherapy	*n*	Death	Long survival	Long survival rate
Poorly	Yes	2	0	2	100.0%
	No	16	7	9	56.3%
	All	18	7	11	61.1%
Moderately and well	Yes	5	2	3	60.0%
	No	50	5	45	90.0%
	All	55	7	48	87.3%
	Total	73	14	59	80.8%

## 3 讨论

新的肺癌分期^[5]^已经于2009年7月在美国洛山矶第13届世界肺癌大会上提出。经过12年的大量肺癌临床数据分析，在分期中着重指出病灶大小在NSCLC中起着重要预后作用，并指导临床NSCLC分期，将≤2 cm划归为T1a并指出侵犯胸膜的归为T2，即Ⅰ期中的Ⅰb期。并且提出侵犯脏层胸膜弹力层的概念以研究胸膜侵犯的亚型。本研究通过对T1a早期NSCLC的研究发现，侵犯胸膜对病灶≤2 cm的Ⅰ期NSCLC预后影响有限，是否侵犯胸膜对患者长期生存影响不大，对该类患者的分期影响亦不明显。而相关文献报道胸膜侵犯影响患者预后生存^[6, 7]^，指出胸膜侵润和肿瘤大小无关, 和纵隔淋巴结转移有关，和长期生存有关，是预后的独立的因素。Hyo^[8]^在对脏层胸膜侵袭中也指出其可作为独立的预后因素，但这些研究并未局限在癌灶≤2 cm的Ⅰ期NSCLC。本文回顾性研究中并未发现病灶≤2 cm的T1a早期NSLCL胸膜侵犯对长期生存的影响具有统计学意义，Pisters^[9]^在文献中也指出其并不是独立预后因素。相反肿瘤的低分化或中低分化程度严重影响患者预后，这与Ou研究的结果相仿^[10]^。我们可以看到，低分化是患者生存的独立因素，影响患者的长期生存。而胸膜侵犯对癌灶≤2 cm早期患者的研究结果和前人的研究结论相反，可能是因为既往的研究并未将影响预后的重要因素直径进行分类讨论。

系统性纵隔淋巴结清扫在各期NSCLC根治性切除中已达成共识，可提供分期的诊断，有助于患者预后分析，为其它治疗提供依据^[11]^。相关研究也指出纵隔淋巴结系统切除对于Ⅰ期NSCLC也同样具有较为积极地预后意义^[12, 13]^，但是在病灶≤2 cm的Ⅰ期NSCLC中，Ma^[14]^和Takizawa^[15]^通过将直径≤2 cm的NSCLC分成纵隔淋巴结系统清扫和采样术两组，并没有发现两者的5年总体生存率和无病生存率有区别，本研究也发现系统淋巴结清扫和纵隔淋巴结采样两组之间患者长期总体生存并无明显区别，Okada^[16]^甚至提出对所有无论直径大小的Ⅰ期患者采用有选择性的淋巴结切除，这种理念略显偏激，随后也被大样本的临床研究否定。

同时术后辅助化疗在早期NSCLC中存在很大的争议，多数学者认为化疗对所有Ⅰ期NSCLC没有疗效^[17]^，不是患者长期生存的必要因素^[12, 18]^甚至对Ⅰ期肺癌病人术后辅助化疗增加致死性的危险可能^[9]^。本篇文章中由于化疗样本量较少，多因素分析发现T1a的Ⅰ期NSCLC也得出了术后辅助化疗对于该类患者的长期生存并没有统计学意义。在早期肺癌中，化疗对肺癌的短期无病生存起到一定控制作用^[15]^，但对长期生存并无影响^[9, 19]^。根据影响患者预后的因素分类，分析辅助化疗对早期肺癌的影响，我们可以从表 2中发现，虽然对低分化组化疗只有2组，但是都得到长期生存，相反未化疗的16例长期生存较差，只有9例长期生存（生存率为56.3%），而对中高分化组的5例化疗研究，其长期生存也只有3例，未行术后辅助化疗中高分化组的患者生存率达到90%。由此可见，中分化程度以上的早期T1a的Ⅰ期NSCLC，化疗没有意义，因为具有较高的生存率，临床上认为手术可以根治。

根据肺癌个体化治疗原则，我们认为对于T1a的Ⅰ期NSCLC患者如影像学无明显纵隔淋巴结肿大的患者，可行纵隔淋巴结采样的方式达到肺癌根治，这样有利于减少术后渗出，减少住院时间；而患者的病理类型、是否侵犯脏层胸膜、病理类型、中分化、高分化、中高分化程度并不是病灶≤2 cm的Ⅰ期NSCLC预后因素，临床上特别是侵润脏层胸膜的患者可能不是病灶≤2 cm的Ⅰ期NSCLC运用术后辅助化疗的指征，而对于中分化以下程度的T1aN0M0期NSCLC由于生存率较低，术后辅助化疗可能有用。

本研究提出了分化程度是影响早期肺癌预后的独立因素，同时证实纵隔淋巴结清扫并非在所有早期患者特别是T1a Ⅰ期NSCLC中均有必要，也得出和国际上对Ⅰ期肺癌是否行术后辅助化疗较为不肯定的意见，但是由于是本研究为回顾性研究，样本量不大，且影响肺癌的因素是多样的，因此本文的结论需要进一步研究证实，而从临床TNM分期决定NSCLC病人特别是早期患者的预后也有一定的不足之处，希望能从微观分子标记物角度将NSCLC预后差的病人区别出来以便行个体化治疗。
